# Dr. Joshua Tucker

**Published:** 1882-02

**Authors:** 


### OBITUARY.

At the monthly meeting of the American Academy of Dental Science held in Boston, January 4th, 1882, the following resolutions were unanimously adopted :

*Resolved*, That the American Academy of Dental Science has received with sincere sorrow the intelligence of the death of our late beloved friend and associate, Dr. Joshua Tucker, of Boston, an Honorary Member and former President, of this Society. He departed this life on November 7th 1881, aged 81 years and 3 months.

*Resolved*, That in the decease of Dr. Tucker, this Academy has been called to mourn the loss of one of its most valued members, one whose ability, integrity, genial manner, and kindness of heart, endeared him to all whose privilege it was to be associated with him. By his death another distinguished name is added to the list of those early pioneers in dental science, who have finished their earthly labors and passed on to the land of rest and immortality, and whose memory the profession will ever delight to honor and cherish.

*Resolved*, That this Academy will never forget the deep interest and untiring devotion which our late friend invariably brought to the performance of his duties during his long and successful professional life, embracing a period of more than fifty years. Dr. Tucker was one of the first in this country to take a high stand in the practice of his profession, which he always maintained; and throughout his whole career his actions were always characterized by an enlightened sense of the responsibilities which rested upon him both in his relations with the profession and with those who were placed under his care and treatment, thus proving his constant desire to be faithful to the trust reposed in him. His patients as well as the profession will hold him in grateful remembrance.

*Resolved*, That our sympathies and condolence are extended to his widow and other relatives in their bereavement, and we would, also, rejoice with them that he was permitted to live to such an advanced age, and that he has left so beautiful a record of a long and useful life.

*Resolved,* That these resolutions be entered upon the records of the Academy, and that the Secretary be instructed to transmit a copy of the same to the widow of the deceased, also to the dental and medical journals for publication.

George T. Moffatt,
Jacob L. Williams,
Edward N. Harris,
*Committee.*

